# Synthesis of relevant information around non-core domains to support Multi-Criteria Decision Analysis (MCDA) for decision making

**DOI:** 10.3205/hta000139

**Published:** 2024-03-27

**Authors:** Juan Carlos Bayón-Yusta, Asun Gutiérrez-Iglesias, Lorea Galnares-Cordero, Iñaki Gutiérrez-Ibarluzea

**Affiliations:** 1 Basque Foundation for Health Innovation and Research (BIOEF), Barakaldo, Spain; 2 Osteba, Basque Office for HTA, Barakaldo, Spain; 3 Ministry for Health, Basque Government, Vitoria-Gasteiz, Spain

**Keywords:** Health technology assessment, Nominal group technique, consensus, Multi-criteria decision analysis

## Abstract

Countries fundamentally base macro and micro decision making in the field of health on economic considerations, the budgetary impact of technologies being a major criterion. Nevertheless, the value of the technology of interest and its dimensions are more complex if we seek to take decisions based on the value itself. The use of structured and explicit approaches that require the assessment of multiple criteria that reflect the dimensions of this value may significantly improve the quality of the decision making. Multi-criteria decision analysis (MCDA) is a complementary decision-making tool that is able to systematically incorporate dimensions or domains such as ethical, organisational, legal, environmental and social considerations, as well as costs and benefits of medical interventions, together with the distinct perspectives of the interested parties. The objective of this article is to propose the implementation of analysis of non-core domains, in reports of Health Technology Assessment (HTA) agencies/units. To assess the scientific evidence on MCDA techniques a systematic review was conducted using structured searches in biomedical databases and websites of various HTA organisations. A consensus group was held using the nominal group technique and involving users of healthcare services, providers, managers and academics. Complementary, a survey was sent to HTA agencies to ascertain the degree of implementation of MCDA in their methods. 42 articles reporting the use of non-core criteria for the assessment of health technologies were included in the analysis. From these articles, a total of 216 non-core criteria were retrieved and categorised into domains by the researchers, and of these, 56 were classified as socioeconomic, 59 as organisational, 10 as legal, 8 as environmental and 47 as ethical, while 36 were considered to relate to other domains. The consensus group, based on the 216 non-core criteria obtained from the systematic review, proposed, and defined 26 criteria that participants considered necessary for decision making in healthcare. The consensus group did not consider that any of the domains should be given more weight than others or that any individual criteria should dominate. These approaches can serve as a framework of reference for a well-structured systematic discussion concerning the basis of individual criteria and the evidence supporting them.

## Introduction

Decision-making regarding health, which is often complex and multifaceted, requires careful assessment of existing health technologies and their prospects in a given context, as well as the use of multiple criteria to evaluate available alternatives [1].

Some countries currently support macro and meso decision-making in health mainly on economic aspects, based on the comparison of the costs and the benefits to healthcare services [2]. In most cases, the Anglo-Saxon model, based on cost-utility analysis, is used for this comparison, which is widely accepted because QALYs are assumed to be an objective measure for comparing health technologies. However, the decision is not always justified as the sole criterion in all cases.

Apart from the costs and healthcare benefits, there are other aspects/dimensions to be taken into account in the decision-making process: the degree of innovation of new technology, acceptability and accessibility, as well as ethical, organisational, legal, environmental and social aspects, among others. In addition, the perspectives of each of the stakeholders in the implementation or exclusion of health technologies must be included [3]. 

The use of structured and explicit approaches that require the evaluation of multiple criteria which allow these different perspectives to be incorporated, such as multi-criteria decision analysis (MCDA), can significantly improve the quality of decision-making [1].

The advantages that these models can bring to decision-making seem clear, as they cover all the domains that provide evidence and that influence whether decisions are accepted. They can be explicitly prioritised and respond well to public accountability. However, for such models to work they must receive evidential inputs from all the domains to be considered, otherwise they will be partial and biased. Although there are structured models for approaching decision-making such as the EVIDEM (Evidence and Value: Impact on Decision Making) framework [1], the local Health Technology Assessment (HTA) framework from the University of Calgary [4] or the MEAT (Most Economic Advantageous Tender) framework [5], other analyses that support areas such as ethical, legal, social, cultural, organisational, or environmental impact assessment have not been standardised. Failure to explore these analyses in depth would leave any attempt at decision-making incomplete. Therefore, the aim of this article is to propose the implementation of non-core domain analyses (ethical, legal, social, cultural, organisational, or environmental impact) in the reports of HTA entities in order to respond to MCDA decision-making models.

## Methods

### Systematic review of the evidence

A systematic review of the evidence on MCDA models using non-core criteria for decision-making in the incorporation, modification or exclusion of health technologies was carried out.

The search was done in the biomedical databases Medline (PubMed) and Embase (OVID), in selected databases (Web of Science) and nursing databases (CINAHL), as well as in different Health Technology Assessment (HTA) organisations. The references of the included papers were also reviewed manually.

The search strategy included the following free and/or controlled language terms: Multi Criteria Decision Analysis or MCDA, among others. 

Finally, the search was updated to identify new studies before the final edition of this article.

Relevant articles based on their title and abstract were selected by two independent reviewers for full-text reading based on the following inclusion and exclusion criteria:

- Original articles, systematic reviews and procedural guidelines published in peer-reviewed journals up to October 2017 in English or Spanish, which developed MCDA models based on domains or "non-core" criteria to inform on decision-making in Health Technology Assessment were included.
- Articles, systematic reviews or procedural guidelines that did not include MCDA models or use these models to support decisions in Health Technology Assessment and which, even when developing MCDA models for this reason, only relied on core domains were excluded. In addition, papers published on websites, communications given at conferences, letters to the editor, editorials and commentaries were also excluded.

The data from the selected peer-reviewed articles were extracted using tables designed "ad hoc" in which the following were recorded: the author of the article, the domains and criteria analysed, their definition and the health technology assessed. The non-core criteria extracted were classified and grouped in a table by two researchers within the following domains: social (socio-economic), organisational, legal, environmental, ethical and others. 

As the research question addressed is non-clinical, it was not considered necessary to assess the methodological quality of the evidence.

### Consensus technique nominal focus group

A nominal consensus group composed of health care consumers, health care providers, managers and academics, etc. was formed to discuss the criteria that should form part of each of the so-called "non-core" domains. 

The consensus group meeting was organised into four phases:

1. The generation of idea/criteria phase. The group coordinator handed out the "non-core" criteria, previously divided into domains by the researchers. The members of the group could suggest new criteria if they considered that they needed to be included.
2. The recording of ideas/criteria phase. A member of the research group noted down the criteria submitted for each domain by each member of the focus group. The criteria were required to be thorough, not redundant or duplicated, and independent. Each member of the group had three turns per domain to present the criteria they had written down.
3. The discussion phase (recorded with the members' permission). The purpose of each of the recorded criteria was discussed and any queries about their significance were clarified. This meant that the criteria could be grouped together to form more extensive criteria, moved from one domain to another, or discarded. The result was one set of criteria for each domain. As some of the criteria obtained were later deemed to be somewhat inoperable by the research team, the team redid them and emailed them to the members of the focus group for their approval and/or modification.
4. The voting phase (conducted by webmail). The criteria for each domain were sent to each member of the group to vote on. A nominal scale of 10 to 1 (10 being the most important criteria for the domain and 1 the least important) was used. The criteria with an average score of six or above were selected.

The final criteria for each domain were defined by the research team. The list of criteria and their definitions was sent by webmail to the members of the consensus group for review and modification. Once all the contributions were received, the research team drafted the final list of criteria and their definitions, which was the nominal focus group's final product.

Finally, the consensus group was asked (via email) to reflect on how the selected criteria should be dealt with within the MCDA models.

A questionnaire was also developed to ascertain the level of development and depth of non-core domains in national and international HTA agencies. This questionnaire was sent to the agencies of the Spanish HTA Network (https://redets.sanidad.gob.es) and the international INAHTA network (https://www.inahta.org).

## Results

### Bibliographic search

**Figure 1:**
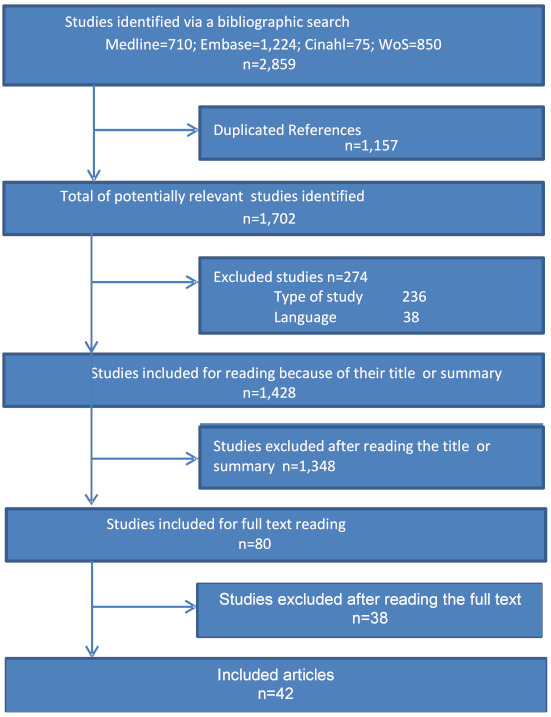
Flow chart

The bibliographic search identified 42 articles for analysis and (Figure 1 (Fig. 1)) search update, which was carried out in May 2018, did not identify any further relevant articles to be included in the study. Of the 42 studies included, four were systematic reviews and the remaining articles developed general MCDA models for decision-making in HTA.

The MCDA models were observed to be the most frequently used in high-income countries in the European framework and Canada, although middle or low-income countries such as Thailand, Morocco, Iran, Ghana or Colombia also used them occasionally. 

The MCDA models analysed evaluated medicines (off-patent, orphan or new), public health programmes (HIV, obesity), medical devices (cardiac sensors, surgical assistance robots, radicular screws for lumbar arthrodesis) and general treatments (oncology, Turner's syndrome, Lyme disease). 

Most of the studies were conducted on a national level, although some were also carried out on a regional level (Catalonia, Lombardy) or within a hospital. 

Of the 42 articles analysed, 216 non-core criteria were extracted, 56 of which were included in the social (socio-economic) domain, 59 in the organisational domain, 10 in the legal domain, 8 in the environmental domain, 47 in the ethical domain and 36 in others.

### Results of the nominal focus group

9 out of 10 people attended the nominal group meeting. The person who did not attend was informed about the meeting and was sent the documents that had been worked on, participating in the voting and criteria definition phase.

The criteria for each domain and their definition as well as the final product elaborated by the nominal group are reflected in table 1(Tab. 1), table 2(Tab. 2), table 3(Tab. 3), table 4(Tab. 4), table 5(Tab. 5) and table 6(Tab. 6).

The focus group was asked to reflect on how the selected criteria for each domain should be dealt with. In their view, they should all be taken into account in health decision-making when introducing new health technologies. However, one person pointed out that this may add to the difficulties in developing MCDA models.

Regarding the question of whether or not to weight the importance of the chosen criteria, the majority of the group sees no difference among them, considering them all to be of equal importance. However, one person thought that more importance should be given to the ethical domain criteria, and another believed that the option of weighting them should be left open depending on the health programme to be evaluated. Nonetheless, the latter may present certain problems as there are some disadvantages, such as a lack of homogeneity or uniformity in the evaluation process.

### Results of the questionnaire sent to RedETS and INAHTA

At a Spanish level, none of the HTA organisations belonging to RedETS have had any experience or used MCDA models for HTA. At an international level, it is known that some of them are using MCDA models for decision-making. Of the 9 responses received (16.4 %), 4 health technology assessment organisations (CDE, Taiwan; IQWiG, Germany; MaHTAS, Malaysia; ZINL, The Netherlands) (7.29 %) indicate that different experiences and MCDA models have been used in their country or region. However, only 3 of them (CDE, Taiwan; MaHTAS, Malaysia; ZINL, The Netherlands) have used them for HTA. Further, in the INAHTA Congress of 2018, 34 of the agencies present confirmed that they had not implemented MCDA approaches.

## Discussion

This article aims to respond to the need to incorporate qualitative/contextual criteria, through systematic and transparent processes, into health decision-making for the introduction, modification and elimination of health technologies. To this end, a proposal has been drawn up for the implementation of the analysis of non-core domains (ethical, legal, social, organisational, environmental and others) in the reports of HTA bodies and to provide information that will allow a response to the MCDA decision-making models in their different context-dependent frameworks.

In accordance with ISPOR's good practice guidance for conducting MCDA to support health decision-making [6], in the proposed MCDA model, firstly, the corresponding criteria were selected and structured through a systematic search of the published literature on MCDA models in which "non-core" criteria were used for decision-making in the incorporation, modification or exclusion of health technologies. Secondly, a nominal focus group was formed to discuss these criteria with the aim of selecting and defining those considered most important for the domains analysed. The mathematical elements (rating the alternatives, weighting the criteria and calculating an aggregate score) were not incorporated because the aim is to develop a deliberative process that encourages discussion about the selected criteria, thus complying with the broader definition given by ISPOR for MCDA: "methods that aid deliberative discussion using explicitly defined criteria, but without quantitative modelling" [7].

Why is a deliberative process being proposed? As Culyer, 2014 [8] pointed out, a deliberative process is participatory and is often followed by a period of consultation with relevant stakeholders and involves obtaining and combining various types of evidence in order to arrive at an evidence-based judgement. Furthermore, Poulin also pointed out [9] that if individual criteria are not weighted to reflect their importance, the relevance of a specific criteria may change depending on the case. Therefore, using a deliberative process in MCDAs to make recommendations for health technology assessment is the most suitable way to provide a guide for systematic discussion and a clear understanding of how each criteria and its related evidence affects the final discussion. Similarly, Jehu-Appiah Younkong and Tanios [10], [11], [12] noted that MCDAs should include a deliberative process to address non-quantitative concerns (evaluations of ethical and social acceptability as well as the complexity of the intervention) and to foster balanced judgements about intervention priorities in order to reach a consensus among stakeholders by facilitating discussion and decisions across jurisdictions, levels of decision-making and perspectives.

In addition, several authors have raised a number of concerns when assigning criteria and weightings in MCDA that support the adoption of the proposed deliberative model. Thus, Phelps et al, 2017 [13] noted that the weight-setting protocol in the analytic hierarchy process may allow for internal inconsistencies and rank revision or change in the hierarchical order of desirability between different decision options. Marsh et al, 2017 [14] pointed out that since weightings reflect trade-offs between degrees of performance on criterion scales, stakeholders tasked with providing them need to keep the range of the scales in mind, so they should not be tendered independently of the range of consequences. 

Another concern that may emerge is how to address potentially divergent weights from different stakeholders [11]. Given the subjective nature of the weightings, these reflect the multifaceted meaning and values determined by stakeholders [15]. It is complicated to assess their face validity without a precise definition of the criteria [14], as these may establish weights in unexpected ways [15] producing unintended consequences.

On the other hand, Garattini et al., and Walker et al., [16], [17] said that it is difficult to predict whether the scores and weights of the main factors stimulate debate among decision makers or strengthen the role of the specialists administering the procedure since greater empowerment is given to the people preparing the scores and weights. The apparent numerical precision of MCDAs can be misleading for decision makers, giving the false impression that the final results are scientifically proven objective numbers [16].

Finally, within the EVIDEM framework, which is a reflective multi-criteria approach designed to support the culture of reasonable decision-making by promoting procedural and substantive legitimacy, it is proposed that contextual criteria be used as a guide to adapt the framework to the decision-making context. Such criteria may remain in the contextual assessment tool for qualitative considerations [18] with their potential impact being reflected qualitatively and thereby affecting the ranking. When the framework is adapted to a specific context, they can also be added to the core MCDA model for quantitative analyses [18]. However, their inclusion in the overall additive equation would render the MCDA model spurious as the assessment of these criteria is subjective and contextual, whereas the MCDA model manifests objective evidence-based impacts of technology [19]. Contextual criteria would need more formal interpretation in the MCDA process since they can sometimes be critical elements for the decision [20]. Furthermore, Walster et al., Goetghebeur et al., and Wagner et al., [20], [21], [22] indicated that MCDA estimates should not be used as a formulaic approach, but as a guide in decision-making to unravel all relevant quantifiable elements, and then consider the impact of other ethical and contextual elements influencing the overall value.

The main limitation of the study is that a nominal consensus group of 10 members may be too small. Clearly, individuals vary in their evaluations, which may be influenced by personal and professional factors, such as experience, role in society and education. This study was not designed to investigate the impact of "non-core" criteria on assessments, but to make a proposal for their implementation in health technology assessment agency reports. However, it included a diversity of stakeholders (health care consumers, health care providers, managers, academics, etc.) in an attempt to engage a wide variety of perspectives. On the other hand, the small size of the nominal focus group facilitated group discussions and the exchange of ideas, which allowed for a more in-depth analysis of the different aspects involved.

## Conclusions

The selected criteria should be considered by HTA entities when compiling and synthesizing information for health decision-making. The consensus group does not consider that any of the domains should be weighted above others or that individual criteria are more preeminent than others. 

Furthermore, it is proposed that all the necessary information gathered in the health technology assessment process to assist in the deliberative decision-making processes should be included. Structured and informed deliberative models offer a clear advantage over closed and non-informed decision-making processes since they render the reasoning behind the final decision explicit and transparent. These models can serve as a referential framework in a systematic and structured discussion based on individual criteria and supporting evidence. 

## Notes

### Funding statement

This research received funding from the Spanish Ministry of Health, Social Services and Equality for the development of the activities of the Annual Work Plan of the Spanish Network of Agencies for the Evaluation of Health Technologies and NHS Benefits, approved in the Plenary Session of the Interregional Council on November 8, 2017 (in accordance with the Agreement of the Council of Ministers of December 1, 2017).

## Acknowledgements

The authors would like to acknowledge the work done by the consensus group participants: Agustín Martínez Berriochoa (health professional), Antonio de Blas de Blas (manager), Emmanuel Giménez García (health economists), José Luis Quintas-Diez (policy-maker), José Cano-Mesías (patients’ representative), Luisa Arteagoitia-González (policy-maker), Pilar Nicolás-Jiménez (academia), Rosana Triviño-Caballero (ethicist), Sergio Márquez-Peláez (HTA professional) Yolanda Menchaca-Barruetabeña (social services professional) and the help in editing and adapting the manuscript to English language by Lesley Lee.

## Conflict of interest

The authors declare that they have no competing interests.

## Figures and Tables

**Table 1:**
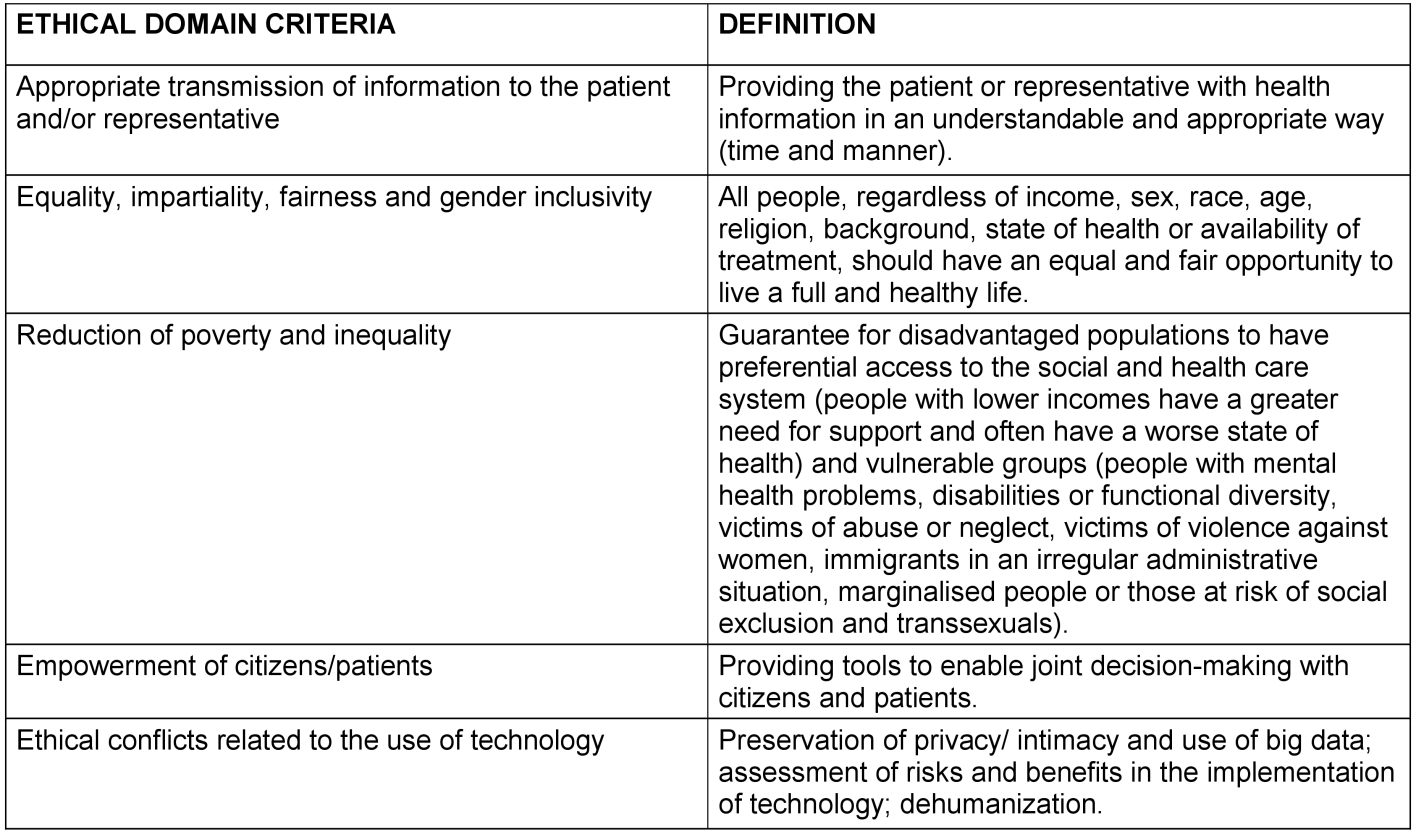
Definition of ethical domain criteria

**Table 2:**
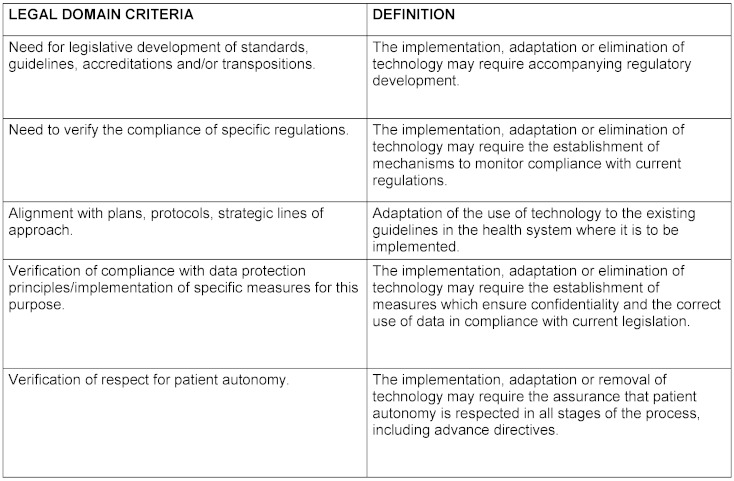
Definition of legal domain criteria

**Table 3:**
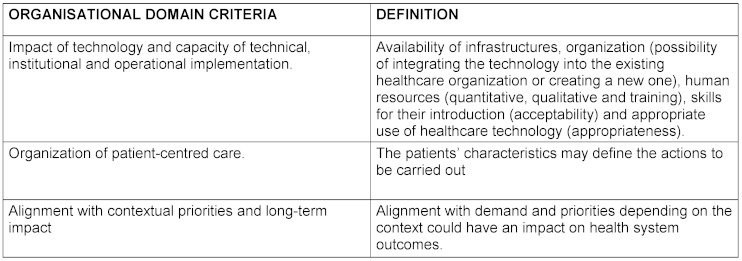
Definition of organisational domain criteria

**Table 4:**
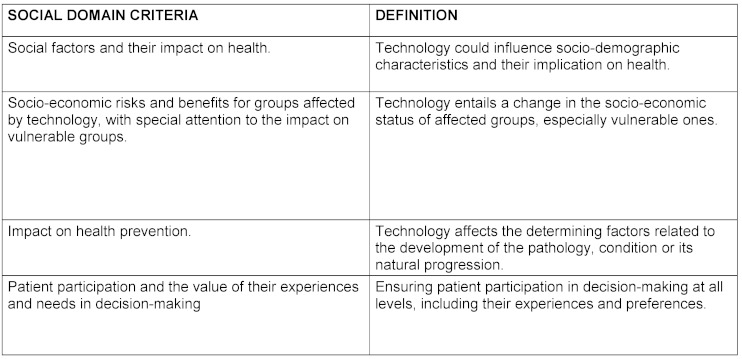
Social domain criteria

**Table 5:**
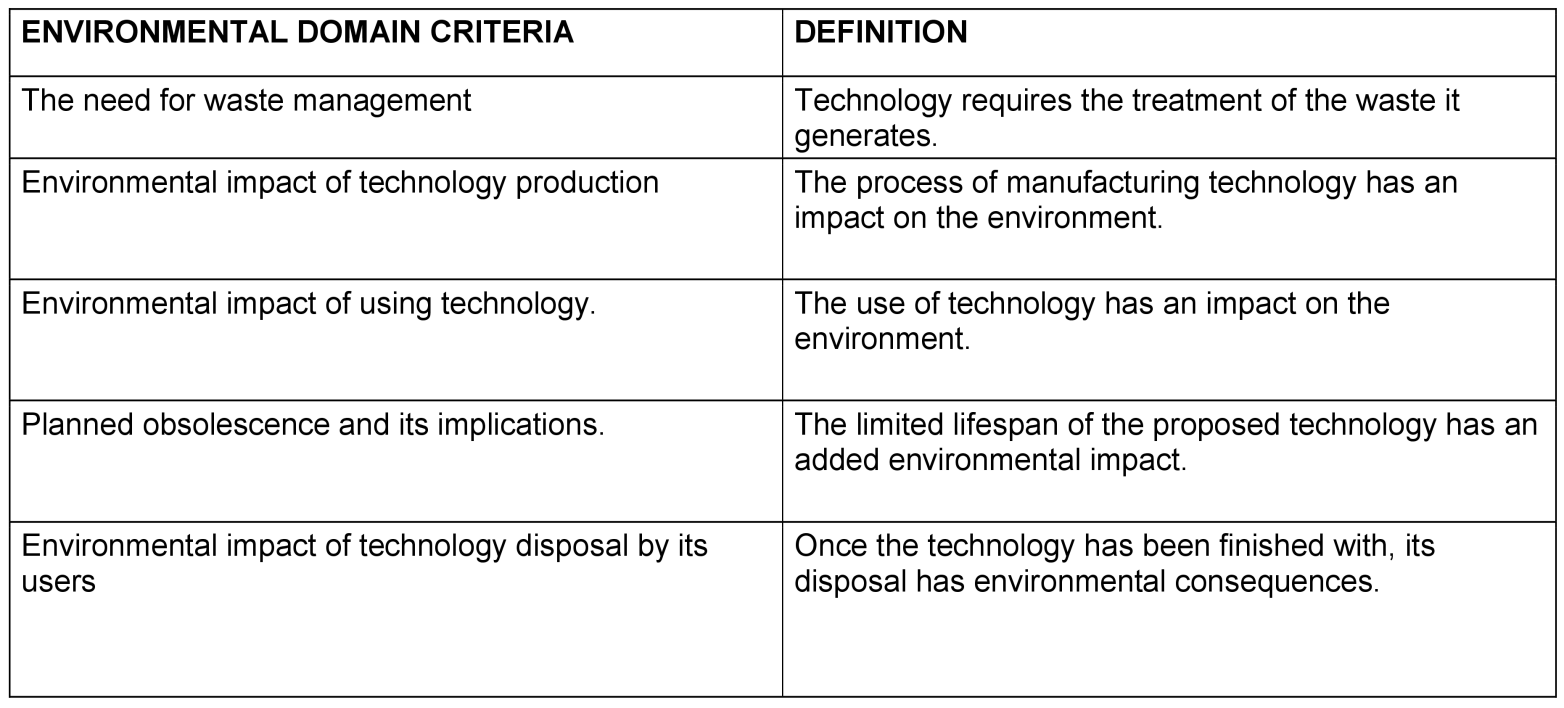
Environmental domain criteria

**Table 6:**
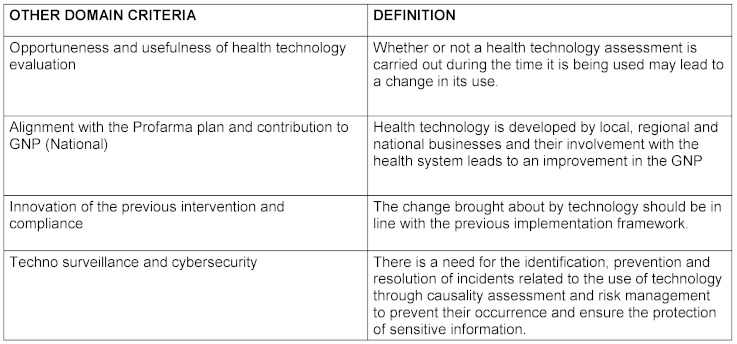
Other domain criteria

## References

[1] Goetghebeur MM, Wagner M, Khoury H, Levitt RJ, Erickson LJ, Rindress D (2012). Bridging Health Technology Assessment (HTA) and Efficient Health Care Decision Making with Multicriteria Decision Analysis (MCDA): Applying the EVIDEM Framework to Medicines Appraisal. Medical Decision Making.

[2] van Velden ME, Severens JL, Novak A (2005). Economic evaluations of healthcare programmes and decision making: the influence of economic evaluations on different healthcare decision-making levels. Pharmacoeconomics.

[3] van der Wilt GJ, Bloemen B, Grin J, Gutierrez-Ibarluzea I, Sampietro-Colom L, Refolo P, Sacchini D, Hofmann B, Sandman L, Oortwijn W (2022). Integrating Empirical Analysis and Normative Inquiry in Health Technology Assessment: The Values in Doing Assessments of Health Technologies Approach. Int J Technol Assess Health Care.

[4] Poulin P, Austen L, Scott CM, Poulin M, Gall N, Seidel J, Lafrenière R (2013). Introduction of new technologies and decision-making processes: a framework to adapt a Local Health Technology Decision Support Program for other local settings. Med Devices (Auckl).

[5] MedTech Europe (2018). Most Economically Advantageous Tender Value-Based Procurement (MEAT VBP): Initiative overview.

[6] Marsh K, Ijzerman M, Thokala P, Baltussen R, Boysen M, Kalo Z (2016). Multiple Criteria Decision Analysis for Health Care Decision Making: Emerging good practices: Report 2 of the ISPOR MCDA Emerging Good Practices Task Force. Value in Health.

[7] Thokala P, Devlinb N, Mars K, Baltussen R, Boysen M, Kalo Z (2016). Multiple Criteria Decision Analysis for Health Care Decision Making An Introduction: Report 1 of the ISPOR MCDA Emerging Good Practices Task Force. Value in Health.

[8] Culyer AJ (2014). Where are the Limits of Cost-Effectiveness Analysis and Health Technology Assessment. J Med Assoc Thai.

[9] Poulin P, Austen L, Scott CM, Waddell CD, Dixon E, Poulin M (2013). Multi-criteria development and incorporation into decision tools for health technology adoption. J Health Organ Manag.

[10] Jehu-Appiah C, Baltussen R, Acquah C, Aikins M, d’Almeida SA, Bosu WK (2008). Balancing equity and efficiency in health priorities in Ghana: the use of multicriteria decision analysis. Value Health.

[11] Youngkong S, Baltussen R, Tantivess S, Mohara A, Teerawattananon Y (2012). Multicriteria decision analysis for including health interventions in the universal health coverage benefit package in Thailand. Value Health.

[12] Tanios N, Wagner M, Tony M, Baltussen R, van TJ, Rindress D (2013). Which criteria are considered in healthcare decisions? Insights from an international survey of policy and clinical decision makers. International Journal of Technology Assessment in Health Care.

[13] Phelps CE, Madhavan G (2017). Using Multicriteria Approaches to Assess the Value of Health Care. Value Health.

[14] Marsh KD, Sculpher M, Caro JJ, Tervonen T (2018). The Use of MCDA in HTA: Great Potential, but More Effort Needed. Value Health.

[15] Hoshikawa K, Ono S (2017). Discrepancies between multicriteria decision analysis-based ranking and intuitive ranking for pharmaceutical benefit-risk profiles in a hypothetical setting. J Clin Pharm Ther.

[16] Garattini L, Padula A (2018). Multiple criteria decision analysis in health technology assessment for drugs: Just another illusion?. Appl Health Econ Health Policy.

[17] Walker A (2016). Challenges in Using MCDA for Reimbursement Decisions on New Medicines?. Value Health.

[18] Wagner M, Khoury H, Willet J, Rindress D, Goetghebeur M (2016). Can the EVIDEM Framework Tackle Issues Raised by Evaluating Treatments for Rare Diseases: Analysis of Issues and Policies, and Context-Specific Adaptation. Pharmacoeconomics.

[19] Radaelli G, Lettieri E, Masella C, Merlino L, Strada A, Tringali M (2014). Implementation of EUnetHTA core Model(®) in Lombardia: the VTS framework. Int J Technol Assess Health Care.

[20] Wahlster P, Goetghebeur M, Kriza C, Niederlander C, Kolominsky-Rabas P (2015). Balancing costs and benefits at different stages of medical innovation: a systematic review of Multi-criteria decision analysis (MCDA). BMC Health Serv Res.

[21] Goetghebeur MM, Wagner M, Khoury H, Rindress D, Gregoire JP, Deal C (2010). Combining multicriteria decision analysis, ethics and health technology assessment: applying the EVIDEM decision-making framework to growth hormone for Turner syndrome patients. Cost Eff Resour Alloc.

[22] Wagner M, Khoury H, Bennetts L, Berto P, Ehreth J, Badia X, Goetghebeur M (2017). Appraising the holistic value of Lenvatinib for radio-iodine refractory differentiated thyroid cancer: A multi-country study applying pragmatic MCDA. BMC Cancer.

